# Secoiridoids Metabolism Response to Wounding in Common Centaury (*Centaurium erythraea* Rafn) Leaves

**DOI:** 10.3390/plants8120589

**Published:** 2019-12-11

**Authors:** Jelena Božunović, Marijana Skorić, Dragana Matekalo, Suzana Živković, Milan Dragićević, Neda Aničić, Biljana Filipović, Tijana Banjanac, Branislav Šiler, Danijela Mišić

**Affiliations:** Institute for Biological Research “Siniša Stanković”-National Institute of Republic of Serbia, University of Belgrade, Bulevar despota Stefana 142, 11060 Belgrade, Serbia; jelena.boljevic@ibiss.bg.ac.rs (J.B.); perunica@gmail.com (D.M.); suzy@ibiss.bg.ac.rs (S.Ž.); mdragicevic@ibiss.bg.ac.rs (M.D.); neda.anicic@ibiss.bg.ac.rs (N.A.); biljana.nikolic@ibiss.bg.ac.rs (B.F.); tbanjanac@ibiss.bg.ac.rs (T.B.); branislav.siler@ibiss.bg.ac.rs (B.Š.)

**Keywords:** common centaury, *Centaurium erythraea* Rafn, wounding, secoiridoid glucosides, biosynthesis, transcription factors

## Abstract

*Centaurium erythraea* Rafn produces and accumulates various biologically active specialized metabolites, including secoiridoid glucosides (SGs), which help plants to cope with unfavorable environmental conditions. Specialized metabolism is commonly modulated in a way to increase the level of protective metabolites, such as SGs. Here, we report the molecular background of the wounding-induced changes in SGs metabolism for the first time. The mechanical wounding of leaves leads to a coordinated up-regulation of SGs biosynthetic genes and corresponding JA-related transcription factors (TFs) after 24 h, which results in the increase of metabolic flux through the biosynthetic pathway and, finally, leads to the elevated accumulation of SGs 96 h upon injury. The most pronounced increase in relative expression was detected for secologanin synthase (*CeSLS*)*,* highlighting this enzyme as an important point for the regulation of biosynthetic flux through the SG pathway. A similar expression pattern was observed for *CeBIS1,* imposing itself as the TF that is prominently involved in wound-induced regulation of SGs biosynthesis genes. The high degree of positive correlations between and among the biosynthetic genes and targeted TFs expressions indicate the transcriptional regulation of SGs biosynthesis in response to wounding with a significant role of *CeBIS1*, which is a known component of the jasmonic acid (JA) signaling pathway.

## 1. Introduction

*Centaurium erythraea* Rafn, which is known as common centaury or European centaury, is a prominent pharmacological plant due to the presence of many different types of biologically active specialized metabolites. Centaury has long been used in traditional medicine for the treatment of digestive disorders, gastritis, and diabetes [1], whereas many other bioactivities of its extracts have been well documented (reviewed in [2]).

The main terpenoid compounds contained in *C. erythraea* aerial parts are secoiridoid glycosides (secoiridoids/SGs), derivatives of secologanin, among which sweroside (4), swertiamarin (5), and gentiopicrin (6) predominate [3,4,5,6]. The biosynthetic pathway of SGs starts with geraniol and continues through a series of reactions, including oxidation, reduction, glycosylation, cyclization, and methylation steps, and via a number of intermediates such are 8-hydroxygeraniol, 8-oxogeraniol, nepetalactol, iridotrial, 7-deoxyloganetic acid, 7-deoxyloganic acid, loganic acid (1), and loganin (2), results with the first secoiridoid compound in the pathway—secologanin (3). Further biosynthetic route from secologanin (3) to sweroside (4), swertiamarin (5), and gentiopicrin (6) is not fully elucidated. In *Catharanthus roseus*, secologanin is a universal precursor of monoterpenoid indole alkaloids (MIAs) [7], and genes that are involved in the biosynthesis of secologanin have been thoroughly studied [8,9,10]. Recently, we have identified genes encoding for enzymes of the SGs biosynthetic pathway up to secologanin in *C. erythraea* leaves transcriptome, including geranyl diphosphate synthase (*CeGPPS*), geraniol synthase (*CeGES*), geraniol-8-oxidase (*CeG8O*), 8-hydrohygeraniol oxidoreductase (*Ce8HGO*), iridoid synthase (*CeIS*), iridoid oxidase (*CeIO*), 7-deoxyloganetic acid glucosyltransferase (*Ce7DLGT*), two candidates for 7-deoxyloganic acid hydrolase (*Ce7DLH1* and *Ce7DLH2*), loganic acid O-methyltransferase (*CeLAMT*), and secologanin synthase (*CeSLS*) [4]. Organ-specific and genotype-dependent regulation of SGs biosynthesis was demonstrated [4].

The constitutive production of extremely bitter secoiridoid glucosides in *C. erythraea*, as a part of defense strategy, provides continuous and efficient protection against pathogens and herbivores. As an integral part of plant defense itself, these chemicals prevent herbivory, most likely by deterring feeding that is based on the bitter taste aversion. The deterrent effects of iridoid glycosides on generalist and non-adapted specialist insect herbivores are well-documented [11,12,13,14,15]. A series of mechanisms directed to heal the tissues and prevent further damage are promptly activated once the leaf tissues are wounded/injured [16,17]. Wounding-induced effects might also occur in response to some other abiotic (wind, storm, strong rain) or biotic factors (anthropogenic intrusion) since plants are sessile organisms, continuously exposed to various unfavorable environmental conditions. Common centaury grows in open, often transient, temporary habitats with cleared areas, such as dry pastures, early successive stages of establishing forest vegetation (e.g., after deforestation), forest edges, roads and paths, slopes or open forests, channel sides, newly built roads, etc. [18,19]. Thus, it is often exposed to mowing, which results in the wounding of stems and leaves, stimulation of lateral shoots growth and branching, and the inducing of changes in overall physiology and biochemistry.

Generally, responses to wounding occur both in the injured tissue (local response) and in the undamaged areas (systemic response) [16,20]. Most of these responses occur after several seconds to minutes, or in some cases even several hours after wounding [21,22,23,24]. They start with generation, following the perception and transduction of specific signals, and lead to an alteration of expression of numerous genes [21,24,25]. Some of these genes are rapidly affected, including genes that are involved in the biosynthesis of jasmonic acid (JA) and ethylene (ET), as well as those that are involved in the general stress responses [21,23,25]. Thereafter, the modulation of expression of genes that are involved in specialized metabolites biosynthesis pathways (e.g., glucosinolates, alkaloids, phenolic compounds) could be noticed, together with the alterations in the primary metabolism (carbohydrate and lipid metabolism, nitrogen assimilation) [17,22,25].

Specialized metabolites biosynthesis is predominantly controlled at the transcriptional level by the action of specific transcription factors (TFs) [26]. It was shown that pathogen or herbivore attacks can lead to transcriptional reprogramming of plant metabolism, which is signaled by JA and controlled by the different transcriptional regulators (TFs). When the JA levels are low, Jasmonate-Zim Domain (JAZ) proteins bind to TF MYC2, as well as to some additional transcription factors, repressing the expression of early JA-responsive genes [27,28]. However, a burst of JA, promoted by wounding or insect attack, is followed by interaction between the JAZ repressor protein and F-box coronatine insensitive 1 (COI1) protein, causing ubiquitination and resulting in the degradation of the JAZ proteins [29]. Afterward, TFs, such as MYC2, are de-repressed, which leads to the induction of the expression of JA-responsive genes that are involved in the biosynthesis of specialized metabolites or downstream TFs [26]. The JA-responsive TFs, basic helix-loop-helix (bHLH) iridoid synthesis 1 and 2 (BIS1 and BIS2), are known as the major activators of the iridoid pathway in *C. roseus* [30,31]. Jasmonate-associated MYC2-like1/2/3 (JAM1, JAM2, and JAM3) TFs are other components of the JA signaling pathway, which are antagonistic and negative regulators of MYC-like TFs and JA responses [32]. In *C. roseus*, enhanced MIA biosynthesis in response to insects and pathogens is controlled by JA [25,29]. Recent findings suggest that MIA biosynthesis in this species is co-regulated by transcriptional cascades: CrMYC2 and CrBIS1/BIS2 [30,33].

Although induced defense mechanisms are energy-demanding [16,17], wound-induced accumulation of specialized metabolites can be utilized as an effective approach for scaling up the production of these bioactive compounds [22,34,35]. Therefore, the presented study aimed to analyze and determine the links between genes that are involved in SGs biosynthetic pathway, JA induced TFs, and the production of SGs in centaury leaves in response to wounding.

## 2. Results and Discussion

The presence of about 90 iridoids, mainly secoiridoid glucosides (SGs), has been documented from 127 investigated species that belong to the *Gentianaceae* family [36]. Sweroside (**4**), swertiamarin (**5**), and gentiopicrin (**6**) are reported as the most abundant secoiridoids from this family, and it is presumed that the biosynthetic pathway leading from **4** to **5** and **6** is universally present in the whole *Gentianaceae* family [36]. In some species, roots are the site of the highest SGs accumulation [37,38], while, in others, including *C. erythraea*, these compounds are more abundant in the above-ground parts [4,39,40,41]. Furthermore, the content of total SGs in *C. erythraea* leaves is reported not to be significantly influenced by the developmental stage [4]. Nevertheless, the production of constitutive specialized metabolites can be influenced by various endogenous and environmental factors [4,26,42], with wounding being one of the latter [22,25,43,44].

### 2.1. Wounding-Induced Alterations in the SGs Content

**Figure 1:**
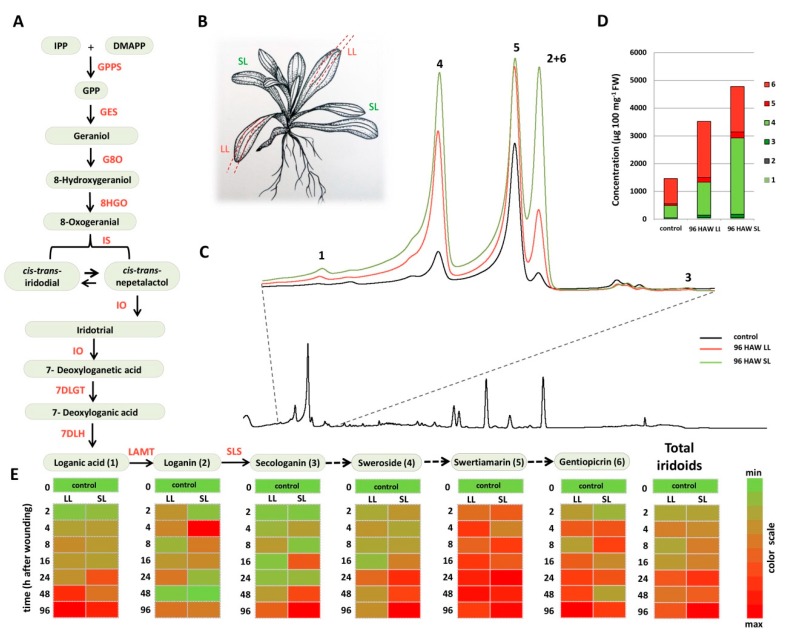
Wounding-induced changes in the accumulation of secoiridoid glucosides in *C. erythraea.* (**A)** The proposed biosynthetic pathway of secoiridoid glucosides (SGs) in *C. erythraea* (modified from [4]). Arrow with a solid line indicates the known enzyme, arrow with dashed line designates unknown enzyme. (**B**) Visualization of the wounding procedure within the experimental set-up: wounded (local leaves–LL) and non-wounded leaves (systemic leaves—SL). (**C**) UHPLC/DAD chromatogram of non-wounded leaves at the beginning of experiment; extracted is the enlarged part of the chromatogram where iridoids and secoiridoids elute. (**D**) Total iridoids content in control leaves and upon 96 h after wounding (HAW), 1—loganic acid, 2—loganin, 3—secologanin, 4—sweroside, 5—swertiamarin, 6—gentiopicrin. (**E**) Heat maps based on the scaled values presenting the relationships between LL and SL of *C. erythraea* at determined time points (h) after wounding (HAW). Scaling was performed for each compound independently. The values are represented by the variation from green (min concentration) to red color (max concentration), as indicated on the color scale. For the interpretation of the references to color in this figure legend, the reader is referred to the web version of this article. Abbreviations: GPPS—geranyl diphosphate synthase, GES—geraniol synthase, G8O—geraniol-8-oxidase, 8HGO-8-hydrohygeraniol oxidoreductase, IS—iridoid synthase, IO—iridoid oxidase, 7DLGT—7-deoxyloganetic acid glucosyltransferase, 7DLH—7-deoxyloganic acid hydrolase, LAMT—loganic acid O-methyltransferase and, SLS—secologanin synthase.

Six major iridoid compounds were initially quantified to assess wound-induced changes within the iridoid biosynthetic pathway—two iridoids (**1** and **2**) and four secoiridoids (**3**, **4**, **5,** and **6**). UHPLC/DAD/(±)HESI-MS^2^ analysis provided high-resolution identification of the iridoid compounds in samples and allowed for their quantification with high accuracy of both highly abundant compounds **4**, **5,** and **6,** and those present in much lower amounts (**1**, **2**, and **3**). The compounds were identified by their UHPLC retention times, UV/VIS, and MS spectra, and by the comparison with the standards and literature data. A UHPLC/DAD chromatogram at λ = 260 nm is presented in Figure 1, with extracted part of the chromatogram where iridoids are eluted. Quantification was performed using the SRM mode of the mass spectrometer (Figure S1), which utilized the two diagnostic fragments of each of the compounds, selected in a PIS (Product Ion Scanning) experiment (Table 1).

The molecular ions of all the targeted compounds, except loganic acid (**1**), were visible in the negative ionization mode as adducts of acetic acid, which was present in the mobile phase (Figure S1, Table 1). The tendency of iridoids to form associated ion products–adducts is common [4,45,46]. Loganic acid (**1**), which eluted at Rt = 2.01 min., displayed pseudomolecular ion [M−H]^−^ at *m/z* 375 and diagnostic MS^2^ fragments [M−C_6_H_10_O_5_−H]^−^ at *m/z* 213 corresponding to the loss of the hexose moiety (−162 Da), and [M−C_6_H_10_O_5_−CH_2_O−H] at *m/z* 168, which is in accordance with some previous studies [46]. Loganin (**2**) showed an acetic acid associated ion [M+CH_3_COOH−H]^−^ at *m/z* 449 and eluted at Rt = 2.88 min. It was possible to lose glucose and subsequently yield MS^2^ ion [M−C_6_H_10_O_5_−H]^−^ at *m/z* 227, while another diagnostic fragment was [^1,4^F]^−^ at *m/z* 127 [4,47]. Secologanin (**3**) with [M+CH_3_COOH−H]^−^ at *m/z* 447 eluted at Rt = 3.66 min. and displayed major MS^2^ fragments [M−C_6_H_10_O_5_−H_2_O−CO−24−H]^−^ at *m/z* 155 resulting from RDA (Retro-Diels–Alder) cleavage and [M−C_6_H_10_O_5_−H_2_O−CO−C_2_O_2_−H]^−^ at *m/z* 123. Pseudomolecular ion [M+CH_3_COOH−H]^−^ at *m/z* 417, which was visible as a peak eluting at Rt = 3.00 min., was assigned to sweroside (**4)**, and it generated major MS^2^ product ions [M−C_6_H_10_O_5_−H]^−^ at *m/z* 195 by the loss of a glucose unit and [C_6_H_12_O_6_−H_2_O−H]^−^ at *m/z* 161. Swertiamarin (**5**) with pseudomolecular ion [M+CH_3_COOH−H]^−^ at *m/z* 433 eluted at Rt = 2.61 min. and its main MS^2^ diagnostic fragments were [C_6_H_12_O_6_−H]^−^ at *m/z* 179, corresponding to deprotonated glucose, and [M−C_7_H_12_O_8_−H]^−^ at *m/z* 149 resulting from the loss of the glucose, H_2_O, and CO_2_ moieties. Compound **6** with [M+CH_3_COOH−H]^−^ at *m/z* 415, eluting at Rt = 2.90 min., was identified as gentiopicrin (Figure S1). Its MS^2^ data showed the fragments [C_6_H_12_O_6_−H]^−^ at *m/z* 179 corresponding to deprotonated glucose, and [M−H−C_8_H_12_O_8_]^−^ at *m/z* 119, which resulted from the loss of glucose, H_2_O, and C_2_O_2_ moieties. Similar fragmentation patterns of compounds **4**–**6** were previously described [45].

The highest increase in total iridoids content was detected at 96 HAW in the SL leaves (Figure 1). The most abundant compound among analyzed iridoids was **4**, which reached 2753 μg 100 mg^−1^ FW (Figure 1). The second most abundant SG in analyzed samples was **6** (~2000 μg 100 mg^−1^ FW), followed by **5** (~200 μg 100 mg^−1^ FW) and **3** (up to 123 μg 100 mg^−1^ FW). Iridoids **1** and **2** reached up to 60 and 4μg 100 mg^−1^ FW, respectively. The content of **1** and four SGs (**3**–**6**) in *C. erythraea* leaves was increased after wounding in a time-dependent manner, showing similar accumulation patterns (Figure 1, heat maps). The accumulation of **5** and **6** started 2–4 HAW (hours after wounding) in both local and systemic leaves, while an increased production of **3** and **4** was observed 16 and 24 HAW. Accumulation of **5** reached a maximum 24 HAW, whereas 3, 4 and 6 reached their maxima 96 HAW (Figure 1). The accumulation of **2** was not time-dependent and wounding-induced and its amount varied in the range of 1–4 μg 100 mg^−1^ FW. In LL and SL, noteworthy changes in total iridoids (predominantly SGs) content were observed from 24 HAW until 96 HAW. Generally, these results are in accordance with some previous studies that demonstrated that wounding-induced accumulation of specialized metabolites occur in both LL and SL leaves, which further supported the hypothesis that wounding stress signals are transmitted throughout the whole plant [16,25,44,48]. As previously mentioned, SGs biosynthesis in *C. erythraea* goes through secologanin (**3**), which is a known precursor for numerous monoterpenoid indole alkaloids (MIAs) [7]. Comprehensive studies in *C. roseus* significantly contributed to the knowledge regarding MIAs biosynthetic pathway and its regulation [8,10,30,31,33]. It has been reported that the MIAs biosynthetic pathway in *C. roseus* is wounding-inducible [25,42,49]. For example, wounding has been found to induce an increase in corynanthe-type MIA accumulation (ajmalicine), whereas iboga MIA (catharanthine) remained unaltered [49]. An enhanced production of specific MIAs in *C. roseus* has been also observed upon mechanical injury by herbivores attack [25]. Thus, the largest increase of strictosidine was recorded 48 h and 72 h after leaves damage by *Manduca sexta,* while, after 72 h, in both local and distal leaves of *C. roseus,* a significant increase of ajmalicene, serpentine, and vindorosine was observed [25]. The accumulation of benzyl isoquinoline alkaloids (BIAs—nuciferine and N-nornuciferine), was significantly increased in the mechanically wounded lotus leaves, whereby fluctuating the accumulation of total alkaloids content in a temporal manner, which varied between wounded and non-wounded leaves, was observed [44]. Increased levels of certain BIAs were also recorded 3 h and 5 h after wounding in the leaves of *Papaver somniferum* L. [50]. Previous results by Alves et al. [51] showed that the highest induction of tropane alkaloid (TA–scopolamine) biosynthesis was recorded 24 h after artificial damage in *Brugmansia suaveolens*, and subsequently decreased to the constitutive level.

### 2.2. Secoiridoids-Related Biosynthetic Genes Expression Profiles in Response to Wounding

The production, accumulation, and distribution of specialized metabolites within the plant are usually related to the expression levels of the biosynthetic genes. We have recently revealed potential candidates for 10 genes encoding for enzymes responsible for the synthesis of secologanin (**3**) in *C. erythraea,* including *CeGPPS*, *CeGES*, *CeG8O*, *Ce8HGO*, *CeIS*, *CeIO*, *Ce7DLGT*, Ce*7DLH*, *CeLAMT*, and *CeSLS* [4]. The expression of these genes is well correlated with the iridoids content and it is organ-specific, genotype-dependent, and mainly MeJA-inducible [4]. Leaves have been highlighted as a major site of SGs biosynthesis and accumulation, and the transcriptional regulation of iridoids biosynthesis has been proposed [4].

**Figure 2:**
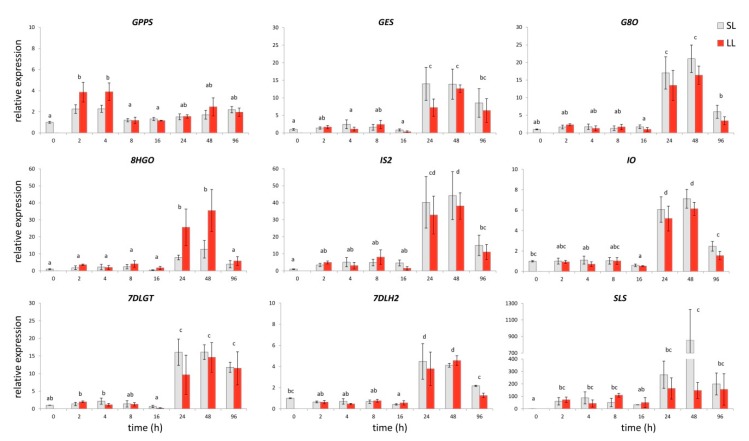
Gene expression of secoiridoid glucosides (SGs) metabolic pathway genes in *C. erythraea* leaves at determined time points (h) after wounding (HAW). The relative expressions of the genes were normalized against *CeEF1* gene as an internal control; non-wounded leaves at 0 HAW were set as a calibrator. Since factorial ANOVA revealed that type of leaves does not significantly contribute to the gene expression, only the effect of time was post hoc tested. In all cases, bars with different letters are significantly different (*p* < 0.05) according to post hoc Tukey’s test. Abbreviations: GPPS–geranyl diphosphate synthase, GES—geraniol synthase, G8O—geraniol-8-oxidase, 8HGO—8-hydrohygeraniol oxidoreductase, IS2—iridoid synthase, IO—iridoid oxidase, 7DLGT—7-deoxyloganetic acid glucosyltransferase, 7DLH2—7-deoxyloganic acid hydrolase, SLS—secologanin synthase, EF1—elongation factor 1, LL—local leaves, and SL—systemic leaves.

We have investigated the time-dependent expression patterns of SGs biosynthetic genes, in both LL and SL, to address transcriptional control of increased SGs accumulation in response to wounding in *C. erythraea*. *CeLAMT* was omitted from the analysis due to its extremely low expression level. Quantitative real-time PCR (qRT-PCR) analysis revealed that nine out of the 10 targeted transcripts showed an increasing trend of expression, not only in LL, but also in the SL (Figure 2). *CeIS1* was the only gene showing no increasing trend of expression after wounding (Figure S2A). In our previous work [4], this gene showed no transcriptional response to the MeJA treatment either. Phylogenetic analysis was performed in order to investigate the evolutionary relationship of *C. erythraea* ISs (CeIS1 and CeIS2) with some of the characterized ISs and progesterone-5-β-reductases (P5BRs) from other species (Figure S2B). CeIS1 evidently subclades with the known P5BRs, while CeIS2 is positioned within the distinct branch of ISs (Figure S2B). Previous studies have shown that *CrIS* in *C. roseus* is co-expressed with the other genes of the MIA pathway, while *CrP5BRs* do not follow the same pattern of expression [52,53]. Thus, *CeIS2* has shown to be a promising candidate based on high sequence similarity to *CrIS* and its co-expression pattern in response to wounding, which looks to be similar to those of the other SGs biosynthetic genes. Although the expression of *CeIS1* is neither MeJA- nor wound-induced, its involvement in constitutive SGs biosynthesis in *C. erythraea* cannot be neglected.

Significantly increased expression levels of the majority of analyzed SGs biosynthetic genes in *C. erythraea* leaves were detected 24 HAW (Figure 2). The exception was observed in the expression pattern of *CeGPPS* which encodes the enzyme that catalyzes the condensation of dimethylallyl diphosphate (DMAPP) and isopentenyl diphosphate (IPP) to geranyl diphosphate (GPP), which is the key precursor of monoterpene biosynthesis. In common centaury, the enhanced *CeGPPS* expression was noticed 2 and 4 HAW. Significantly higher expression levels of *CeGES*, *CeG80, Ce8HGO*, *CeIS2*, *CeIO*, *Ce7DLGT,* and *Ce7DLH2* were recorded 24 and 48 HAW. The highest increase was recorded for *CeSLS* 48 HAW, where its expression was approximately 800 fold higher than in the respective control. No statistically significant differences between LL and SL were determined (Figure 2). Namely, factorial ANOVA revealed that time is the only factor which has a significant impact on the expression of analyzed genes upon wounding (*p* < 0.05), while differences between LL and SL did not contribute to the obtained differences in gene expression (*p* > 0.05). The observed increase in the relative expression of SGs biosynthetic genes was in accordance with the detected increase of total iridoids (principally SGs) content in leaves. The expression patterns of these genes showed a noticeable decrease 96 HAW (Figure 2). Close examination of single genes revealed that *CeSLS* expression, although following the expression pattern of other SGs-related genes, was already elevated 2 HAW in both SL and LL, with statistically significant enhancement 48 HAW. Similar to our results, the expression of *SLS* and *LAMT* genes in *C. roseus* was found to be significantly up-regulated 6 h, 8 h, and 24 h after the *M. sexta* larvae attack and wounding of leaves. However, the expression of genes that were related to the early steps up to the *7DLH* did not display such a high induction [25]. Significant induction of *SLS* transcripts in response to wounding was also recorded in *C. roseus* 6 h after damaging of ~50% of the leaf lamina with a surgical blade [54]. Nishanth et al. [42] also showed that expression level of *SLS* was largely up-regulated in *C. roseus* 24 h after wounding of leaves with a surgical blade, together with some other TIA biosynthesis genes (*STR* and *PX1*).

**Figure 3:**
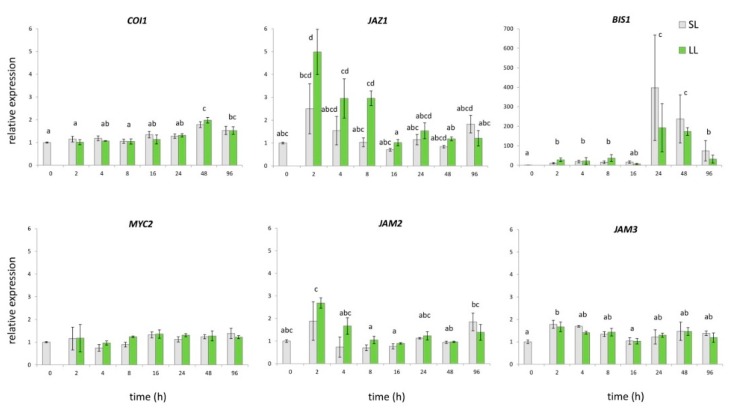
Relative gene expression patterns of selected transcription factors in *C. erythraea* leaves at determined time points (h) after wounding (HAW). The relative expressions of the genes were normalized against *CeEF1* gene as an internal control; unwounded leaves at 0 HAW were set as a calibrator. Since factorial ANOVA revealed that leaf type does not significantly contribute to the expression of *CeCOI1*, *CeBIS1*, *CeJAM2,* and *CeJAM3* transcription factors, only the effect of time was post hoc tested. In these cases, bars with different letters are significantly different (*p* < 0.05) according to post hoc Tukey’s test. Leaf type (SL or LL) was the factor that significantly contributed to gene expression of *CeMYC2.* Factorial ANOVA indicated that time, leaf type, and interaction time x condition significantly contribute to the *CeJAZ1* gene expression. In this case each bar with different letters is significantly different (*p* < 0.05) according to post hoc Tukey’s test. Abbreviations: LL—local leaves, SL—systemic leaves.

Plants act in response to either wounding or herbivore attack by triggering complex signaling pathways, whereas the synthesis and perception of JA and its derivatives are considered to be of key importance [28,29,55,56]. Endogenous JA levels promptly increase upon wounding, in damaged as well as in non-damaged distal tissues [48,55,57]. The increased JA levels are perceived via COI/JAZ co-receptor complex, whereby COI1 ubiquitinates JAZ transcription repressors. Subsequently, JAZ degradation releases JA-responsive transcription factors (TFs) and activates wound-induced gene expression [58]. In *C. roseus,* the expression of MIA structural and regulatory genes are mainly JA-responsive, with COI1, MYC2, and JAZs representing the crucial elements of the JA signaling pathway [33]. Transcription factor CrMYC2, which belongs to the IIIe subgroup of bHLH TF, is a master regulator that activates CrORCAs (directly ORCA2 and ORCA3), the AP2/ERF family transcription factors, thus leading to the induced expression of several MIA biosynthetic genes [29]. In addition to MYC2, another two JA-inducible bHLH transcription factors, BIS1 and BIS2, from clade IVa, have proven to be specifically involved in iridoid branch of MIA biosynthesis in *C. roseus* [30,31]. The overexpression of *CrBIS1* or *CrBIS2* leads to the increased expression of iridoid pathway genes and early MEP pathway genes, as well as to the increased accumulation of TIAs [28,30,31]. Both of the genes are specifically involved in the iridoid branch and are under control of an amplification loop [28]. It is accepted that these two TFs are involved in the regulation of the structural genes expression in *C. roseus* that MYC2/ORCA3 cascade cannot cover [31,39]. Conversely, JAM1 and its homologues, JAM2 and JAM3, are negative regulators of MYC-like TFs and JA responses. These TFs, which belong to the subgroup IIId of bHLHs, most likely interfere with or block the binding of MYC-like TFs to the JA-responsive genes’ promoters [32,56]. Based on the presented studies, we searched for specific transcription factors that might regulate (affect) the expression of SGs biosynthetic genes in common centaury upon wounding. By analyzing *C. erythraea* leaf transcriptome database [59], we identified nucleotide sequences of several transcription factors candidates (*CeMYC2, CeBIS1, CeJAZ1, CeCOI1, CeJAM2,* and *CeJAM3*) for which we expected to be included in wounding-induced regulation of SGs biosynthesis. On the other hand, no putative genes for the ORCA2 and ORCA3 transcriptional factors were found. In addition for BIS2, which has previously been identified in *C. roseus* [31], no candidate genes were retrieved in the *C. erythraea* leaves transcriptome database. It is possible that this functional homologue of BIS1 is specific for the regulation of MIA-related genes downstream of secologanin in *C. roseus*, and it has no homologue in *C. erythraea*. *CeJAZ1* expression was induced 2 HAW, while minor increase in *CeJAM3* and *CeCOI1* expression was noted 2/4 HAW and 48 HAW, respectively, according to the qPCR results (Figure 3). The enhanced expression of *CeBIS1* was detected already 2 HAW, and the max transcript amounts were recorded 24 HAW (Figure 3). Interestingly, factorial ANOVA revealed differences between factors that influence the expression of each of the analyzed *C. erythraea* TFs. To be precise, time was the only factor that has a significant impact on the expression of *CeCOI1*, *CeBIS1*, *CeJAM2*, and *CeJAM3* upon wounding (*p* < 0.05), as it was for all genes encoding for enzymes that are involved in SGs biosynthesis. The type of leaves (local or systemic) was the factor that influenced *CeMYC2* expression, while expression of CeJAZ1 was influenced by time, by the type of leaves, as well as by the interaction of these two factors (Figure 3). The most important difference in the expression of *CeJAZ1* was observed 2 HAW in LL (Figure 3). Similarly, Van Moerkercke et al. [30] reported an increase of *JAZ1* expression upon wounding in leaves, but not in stems of *C. roseus*. JAZs themselves are wound inducible, and *JAZ* genes exhibit different transcription patterns, which indicate that specific JAZ proteins might regulate different TFs and downstream responses to environmental stresses in specific development stages, tissues, or cell types [58,60]. The expression of *CeBIS1* in leaves of *C. erythraea* upon wounding followed the pattern of *CeSLS* expression, both in terms of time course and intensity. According to the obtained results (Figure 3), *CeBIS1* might be indicated as the TF that is positively involved in wounding-induced regulation of SGs biosynthesis. This is in accordance with the previous finding of Van Moerkercke et al. [30], which suggests that overexpression of *BIS1* caused a dramatic increase of loganic acid and of downstream (seco)-iridoid (secologanin) and MIAs in *C. roseus* (strictosidine, ajmalicine, serpentine, and tabersonine). Expression of *BIS2* showed analogous pattern of changes as *BIS1* [31]. Similarly, Kidd et al. [52] correlated detected low iridoid and MIA content with the lowered expression of *BIS1/BIS2* and of several secologanin-related biosynthetic genes that are expressed in IPAP cells.

The intensive elicitation of the plant specialized biosynthetic pathway genes by JA exogenous treatment was well documented and recognized, but the degree to which metabolic pathways are stimulated is species-specific [61]. Moreover, distinct biosynthetic pathways respond differently to JA treatment. Thus, the MVA pathway genes in C. roseus are not induced by BIS1 overexpression or by JA treatment, while some other genes are [30]. In *Medicago truncatula* overexpression of two bHLH jasmonate-inducible TF genes (*TSAR1* and *TSAR2*) activated all of the genes of the MVA (mevalonate) pathway, but did not affect sterol biosynthetic genes. The overexpression of *TSAR1* mainly enhanced nonhemolytic soya saponin biosynthesis, while the overexpression of *TSAR2* specifically increased hemolytic saponin biosynthesis [62]. Cao et al. [61] indicated that the application of methyl jasmonate (MeJA) significantly increases gentiopicroside (GP) biosynthesis by up-regulating the expression of genes related to the IPP (isopentenyl pyrophosphate) pathway in *Gentiana macrophylla*, but not to the secoiridoid biosynthesis pathway. The same authors confirmed that some of these genes were up-regulated (*8HGO* and *GES)*, while two putative encoding genes for G8O have shown different expression patterns: *G10H1* was up-, and *G10H2* was down-regulated [61]. Similarly, MeJA treatment also increased the transcription of *G8O* in the seedlings of *Swertia mussotii* and it was followed by an increase in the swertiamarin content [63]. The expression of *G8O* has been previously described to be sufficient to increase MIA accumulation in *C. roseus* hairy roots [64]. In our previous study on *C. erythraea,* the application of MeJA for 5 and 10 days induced the accumulation of **2** (loganin), **5** (swertiamarin), and **6** (gentiopicrin), while no significant difference between non-treated and MeJA-treated plants in the amount of **4** (sweroside) and **3** (secologanin) was recorded [4]. Simultaneously, the elevated expression for several SGs biosynthetic genes (*Ce8HGO*, *Ce7DLH2*, *CeIO, CeSLS, CeG8O, Ce7DLGT*) was noted five days after MeJA treatment [4]. Within the present research, changes in iridoids content were monitored during four days following the wounding, and it was noted that wounding leads to the noticeable changes in all of the targeted compounds except **2**. Significantly elevated levels of all genes (with the exception of *CeGPPS*) were also detected 24/48 HAW, showing the tendency of slight decrease after 96 h. It would be interesting to perform and analyze simultaneous wounding- and MeJA-elicitation responses at the level of SGs biosynthetic pathway genes expression and MeJA-responsive TFs to obtain a clearer picture of the involvement of JA signaling network in the regulation of this particular biosynthetic pathway, and that is the course of our further work.

**Figure 4:**
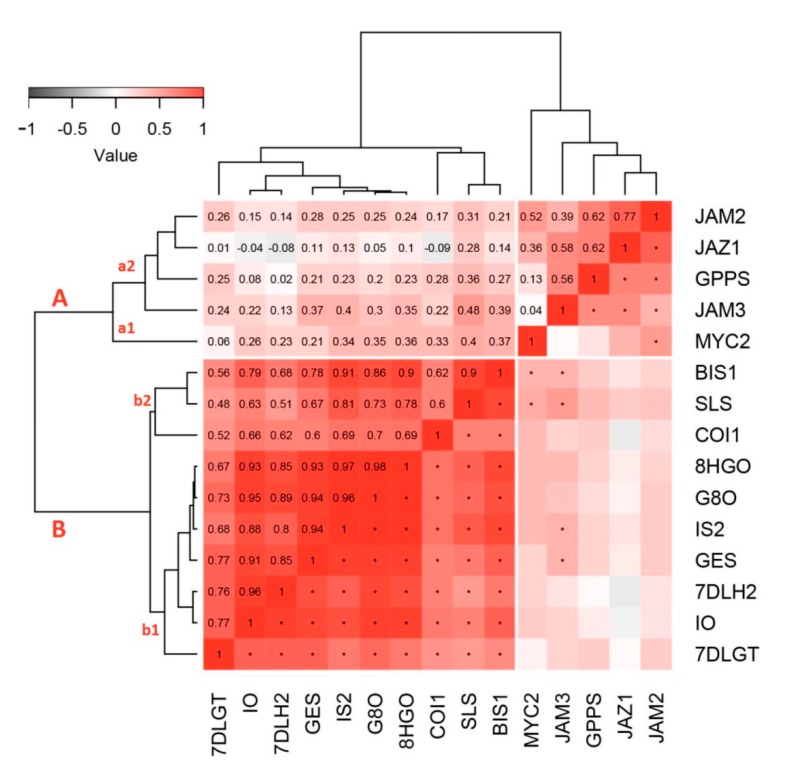
Heatmap of pairwise Pearson correlations based on the relative gene expression of SGs biosynthetic genes and transcription factors. Correlation coefficients are shown in the upper triangle, while statistical significance (*p* < 0.01) is marked with an asterisk at the appropriate positions in the lower triangle. The correlogram is ordered based on hierarchical cluster analysis performed on a distance matrix (1-correlation matrix: minimal distance for absolutely positively correlated and maximal distance for absolutely negatively correlated gene expression). The dendrograms are shown on heatmap sides. All caps represent clusters, while small letters represent sub-clusters. Abbreviations: GPPS—geranyl diphosphate synthase, GES—geraniol synthase, G8O—geraniol-8-oxidase, 8HGO—8-hydrohygeraniol oxidoreductase, IS—iridoid synthase, IO—iridoid oxidase, 7DLGT—7-deoxyloganetic acid glucosyltransferase, 7DLH—7-deoxyloganic acid hydrolase, LAMT—loganic acid O-methyltransferase, SLS—secologanin synthase.

Hierarchical cluster analysis (HCA) was conducted in order to obtain a better insight regarding the linkage among biosynthetic genes and TFs involved in wounding response in *C. erythraea* leaves. Two separated clusters are noticeable based on the presented tree (Figure 4). The first cluster (A) contains majority of TFs (*CeJAM2*, *CeJAM3, CeJAZ1,* and *CeMYC2*), and *CeGPPS* biosynthetic gene. This cluster is separated in two sub-clusters, with the first one (a1) being formed of *CeMYC2*, while other TFs and *CeGPPS* are grouped within the second sub-cluster (a2). All of the other SGs biosynthetic genes and two TFs (*CeBIS1* and *CeCOI1*) are grouped within cluster B and they are also separated into two sub-clusters. The first sub-cluster (b1) is formed of seven biosynthetic genes (*CeGES*, *CeG8O*, *Ce8HGO, CeIS, CeIO, Ce7DLGT*, and *Ce7DLH*), where *Ce7DLGT* is visibly distinguished from the others. On the other hand, *CeSLS* and *CeBIS1*, which displayed similar expression patterns in response to wounding, grouped closely in HCA within the sub-cluster (b2), together with *CeCOI1*. The presented associations between the targeted genes indicate that almost all SGs biosynthetic genes are coordinately expressed in response to wounding and primarily regulated by *CeBIS1* and *CeCOI1,* while *CeGPPS* is mainly under the control of *CeJAZ1* and *CeJAMs.*

A correlation analysis was performed in order to further distinguish TFs that are co-expressed with the biosynthetic genes (Figure 4). Generally positive correlations have been observed between the analyzed biosynthetic genes and TFs, indicating the transcriptional regulation of SGs biosynthesis. *CeBIS1* shows the highest correlation with the biosynthetic genes, in the first place with *CeSLS, CeIS2*, and *Ce8HGO*. A significant, but slightly lower degree of correlation with SGs biosynthetic genes displays *CeCOI1.* Other tested TFs show considerably lower correlation values, whereby no correlation is observed between *CeJAZ1* and *CeJAM2* with other biosynthetic genes, except with *CeGPPS*. A statistically significant positive correlation is found between the expression of *CeJAM3* and *CeGPPS, CeGES, CeIS2*, and *CeSLS,* while *CeMYC2* significantly correlated with two SGs biosynthetic genes: *CeGPPS* and *CeSLS* (Figure 4).

## 3. Materials and Methods

### 3.1. Plant Material

The seeds of *C. erythraea* Rafn were collected at the locality Palja (SE Serbia, GPS coordinates: 42°43′37.64″ N, 22°27′14.07″ E) in July 2010 and further stored at −20 °C, within the Seed collection at the Institute for Biological Research “Siniša Stanković”, University of Belgrade. The centaury seeds were surface-sterilized in 20% commercial bleach for 10 min and then rinsed five times with sterile deionized water. After sterilization, the seeds were then placed on half-strength MS medium [65] containing 20 g L^−1^ sucrose and 7 g L^−1^ agar (Torlak, Serbia). The pH of the medium was adjusted to 5.8 before autoclaving at 121 °C for 25 min. The obtained seedlings were aseptically transferred into 350 mL glass jars that were closed with polycarbonate caps, each containing 70 mL of the ½ MS medium. Leaves from three-month-old *C. erythraea* plants were harvested. Totally, 10 plants/genotypes (P1–P10) were analyzed, as described previously [4], among which the P1 genotype was selected as a SGs high-productive one. This genotype was further subjected to clonal multiplication through root culture. Root tips that were excised from P1 were placed in Erlenmeyer flasks with 50 mL liquid ½ MS medium and grown on a rotary shaker (95 rotations min^−1^) for two months. Regenerated P1 shoots on root explants were transferred into 350 mL glass jars containing 70 mL of solid ½ MS medium, and cultivated for three months. All of the in vitro cultured plants were maintained under long day light regime (16 h light/8 h dark) and temperature of 25 ± 2 °C.

### 3.2. Experimental Setup

Three-month-old clonally propagated P1 plants were used in the experiments to examine the effect of mechanical wounding on SGs production. The mechanical injury was carried out on approximately five leaves per plant, while using scissors to make two small cuts along the leaf nerves (~2 cm) per leaf (illustrated in Figure 1). Leaves from intact plants were used as a respective control. From wounded P1 plants, the damaged (local—LL) and intact (systemic—SL) leaves were separately harvested at 2, 4, 8, 16, 24, 48, and 96 h after wounding (HAW). LL and SL from any of the three individuals were both collected and pooled to obtain three biological replicates. All of the samples were snap-frozen in liquid nitrogen and stored at −80 °C until further use.

### 3.3. Plant Methanol Extracts Preparation

Local (LL) and systemic (SL) leaves of wounded three-month-old *C. erythraea* plants were ground in liquid nitrogen to a fine powder while using a mortar and a pestle. Approximately 100 mg of plant material was extracted with 1 mL 96% methanol (AppliChem, Cheshire, CT, USA). After vortexing for 1 min., the samples were stored at 4 °C and extraction was continued overnight. On the following day, the samples were vortexed for 1 min., extracted for 10 min. in an ultrasonic bath (RK100, Bandelin, Berlin, Germany), and subsequently centrifuged for 10 min. at 8000× *g*. The supernatants were filtered through 0.2 µm cellulose filters (Agilent Technologies, Santa Clara, CA, USA) into glass vials and then stored at 4 °C until further use.

### 3.4. Ultra-High Performance Liquid Chromatography–Tandem Mass Spectrometry (UHPLC–MS/MS) Analysis

For the identification and quantification of SGs in *C. erythraea* methanol extracts, Dionex Ultimate 3000 UHPLC system (Thermo Fisher Scientific, Bremen, Germany) equipped with a triple quadrupole mass spectrometer (TSQ Quantum access max, ThermoFisher Scientific, Basel, Switzerland) was employed. The samples were chromatographically separated on Hypersil gold C18 column (50 × 2.1 mm) with 1.9 μm particle size (Thermo Fisher Scientific, Waltham, MA, USA), thermostated at 30 °C. Mobile phase, consisting of water + 0.01% acetic acid (A) and acetonitrile (B), was eluted according to the gradient previously described in [45]. Acetonitrile was of LC-MS grade (Fisher Scientific, Leics, UK), and ultra-pure deionized water was generated while using Water Purification System (New Human Power I Integrate, Human Corporation, Seoul, Republic of Korea). The flow rate of the mobile phase was set to 0.4 mL min^−1^ and the injection volume to 10 μL. All of the analyses were performed while using three biological replicates. A triple-quadrupole mass spectrometer with a heated electrospray ionization (HESI) source was set to the following parameters: vaporizer temperature 300 °C, spray voltage 4000 V, sheet gas (N_2_) pressure 28 AU, ion sweep gas (N_2_) pressure 1.0 AU and auxiliary gas (N_2_) pressure at 10 AU, capillary temperature 275 °C, and skimmer offset 0 V. Argon was used as the collision gas in the collision-induced fragmentation, and collision energy (cE) was set to 20 eV for all of the targeted compounds. Loganic acid (**1**), loganin (**2**), secologanin (**3**), sweroside (**4**), swertiamarin (**5**) and gentiopicrin (**6**) were quantified while using the selected reaction monitoring (SRM) mode of the instrument, and by tracking two diagnostic MS^2^ fragments of each compound, which were previously defined in product ion scanning (PIS) experiment (Table 1). The identification of the targeted compounds in the samples was additionally confirmed while using DAD analysis (Figure 1), and the data were acquired at λ = 240, 260, and 320 nm. The external standard method was used for the quantification of iridoids and secoiridoids. The preparation of stock-standard solutions was performed by dissolving 1 mg of compounds **1** (Extrasynthese, Genay, France), **2**, **3** (Sigma-Aldrich, Darmstadt, Germany), **4**, **5** (both 98% purity, Oskar Tropitzsch, Marktredwitz, Germany), or **6** (>90% purity, Carl Roth, Karlsruhe, Germany) in 1 mL of 96% methanol. Stock solutions of six standards were mixed to obtain the working standard solution in concentration of 100 μg mL^−1^, which was then diluted with methanol to obtain further calibration levels, up to 5 ng mL^−1^. For each of the calibration curves, the calculation of regression was performed. They all showed excellent linearity with correlation coefficients of r = 0.999, *p* < 0.001. Calculating peak areas was undertaken to obtain the total concentrations of the analyzed iridoids and secoiridoids and they are expressed as μg per 100 mg of plant fresh weight (μg 100 mg^−1^ FW). Xcalibur software (version 2.2) was used for the instrument control, data acquisition, and analysis.

### 3.5. Gene Expression Analysis

The mining of *C. erythraea* leaf transcriptome database [59] has resulted in the identification of nucleotide sequences for SGs biosynthetic pathway genes. Primer pairs for qPCR analysis of 10 SGs metabolic pathway genes (*CeGPPS*, *CeGES*, *CeG8O*, *Ce8HGO*, two *IS* candidates—*CeIS1* and *CeIS2*, *CeIO*, *Ce7DLGT*, *Ce7DLH2, CeLAMT,* and *CeSLS*) were designed while using Primer3Plus software [66], as described by [4]. One more primer pair was designed for *C. erythraea* elongation factor 1 (*CeEF1*), which was used as an endogenous control in qPCR analysis, as well as primer pairs for six transcription factors: *CeCOI1*, *CeJAZ1*, *CeBIS1, CeMYC2, CeJAM2,* and *CeJAM3* (Table S1).

The total RNA was extracted from the collected leaf samples while using a modified protocol of [67]. Approximately 150 mg of each sample was used for RNA isolation, and 1 μg of isolated RNA was treated with DNase I (ThermoFisher Scientific, Waltham, MA, USA) at 37 °C for 30 min. UV absorption spectrophotometer (Agilent 8453 spectrophotometer, Agilent Technologies, Waldbronn, Germany) and Qubit 3.0 fluorometer (Thermo Fisher Scientific, Waltham, MA, USA) were used to determine the total RNA quantity and to test RNA quality. cDNA was synthesized from 300 ng of total RNA while using RevertAid™ First Strand cDNA kit (Thermo Fisher Scientific, Waltham, MA, USA) and oligo-(dT) primers. SYBR Green I (Maxima SYBR Green/ROX Kit, Thermo Scientific, Waltham, MA, USA) was used for the qPCR analysis in a Light cycler QuantStudio 3 (ThermoFisher Scientific, Waltham, MA, USA), according to the manufacturer’s instructions. Thermocycler conditions were 95 °C for 10 min.; 40 cycles of 95 °C for 15 s; 60 °C for 30 s; 72 °C for 30 s; and, final extension at 72 °C for 10 min. The expression levels of targeted genes were calculated according to the 2–ΔΔCt method [68], whereby EF1 gene expression was used as the endogenous control, since this gene showed minimal variation across the control and stress conditions. All of the results are represented as mean value +/− SE from three biological replicates.

### 3.6. Phylogenetic Analysis

Amino acid sequences of the selected iridoid synthases (ISs) and progesterone-5-β-reductases (P5βRs) were aligned with CeIS1 and CeIS2 while using the CLC sequence viewer 8.0 software package (Qiagen, Venlo, The Netherlands). The phylogenetic analysis of the alignment was conducted by the neighbor joining method using the CLC sequence viewer 8.0 with the default settings (Jukes–Cantor protein distance measure and 100 bootstrap replicates).

### 3.7. Statistical Analysis

The relationship between the relative expressions (ddCt) of each of the measured genes was examined while using factorial ANOVA with time and origin of leaves (local and systematic) as the two dependent categorical variables. Box–Cox power transformation [69] was performed to stabilize the response variable prior to the statistical analyses since some of the data were not normally distributed and/or homoscedastic. The analysis of variance was followed by Tukey’s post-hoc test at *p* < 0.05 significance level. Only the factors that were shown to be significant in ANOVA were post hoc tested (Table S2). Pearson correlation between relative gene expressions (ddCt) was used to estimate the co-regulation of gene expression that was visualized by a heatmap. Statistical analysis was performed in the R statistical language [70] while using the packages MASS [71] and gplots [72]. Pearson correlation between relative gene expressions (ddCt) was used to estimate co-regulations of gene expressions which was visualized by a heatmap.

## 4. Conclusions

Based on all of the presented results, it is noticeable that most genes of the SGs biosynthetic pathway in *C. erythraea* are up-regulated upon wounding, whereby the highest increase in the expression level was observed for *CeSLS,* encoding for secologanin synthase, which catalyzes direct cleavage of the cyclopentane ring of loganin to produce secologanin in *C. roseus* [73,74]. The highest amount of transcripts of the majority of SGs biosynthetic genes is reached 24 and 48 HAW, when the responsive TFs showed the highest expression levels and the SGs amount significantly increased. Among the analyzed TFs, the bHLH type *CeBIS1* showed concurrent expression pattern as *CeSLS* and this specific TF, according to the obtained results, has particular significance in wound-induced regulation of SGs biosynthesis in common centaury leaves. In addition, *CeSLS* has been recognized as an important gene/enzyme that might regulate biosynthetic flux through the SGs pathway, and it is probably directly under the control of *CeBIS1* TF. The results further indicate the involvement of the JA signaling pathway in the regulation of SGs biosynthesis, as influenced by wounding. Although differences in the expression levels of other analyzed centaury TFs were not so perceptible, their importance should not be neglected. Although much remains to be investigated in the future, the presented results provide first and significant contribution in understanding the regulatory mechanisms shaping the SGs biosynthetic pathway in common centaury in response to environmental factors. This valuable knowledge could be of great significance for further commercialization and the scaling-up of the SGs production in non-model plant, such as *C. erythraea*, which in turn will provide sustainable sources of these bioactive compounds.

## Figures and Tables

**Table 1:** Iridoids identified in methanol extracts of *Centaurium erythraea* by Ultra-High Performance Liquid Chromatography/DAD/±HESI-MS^2^ (UHPLC/DAD/±HESI-MS^2^) analysis. Peak labels, retention times (t_R_), parent ions [M + CH_3_COOH + H]^+^ *m/z*, MS^2^ fragments used in an SRM (Single Reaction Monitoring) experiment, collision energies (cE), and λ_max_ for each of the compounds are presented.

		UHPLC-MS Data				UHPLC-DAD Data	
Peak No.	Assignment	t_R_ (min)	[M + CH_3_COOH + H]^−^ [*m/z*]	SRM MS^2^ Fragments [*m/z* (Intensity)]	cE (eV)	t_R_ (min)	λ_max_ [nm]
***1***	**Loganic acid ^S,R^**	2.01	375	**213** (100), **168** (<5)	20	1.95	240
***5***	**Swertiamarin ^S,R^**	2.61	433	**179** (100); **161** (15)	20	2.52	240
***2***	**Loganin ^S,R^**	2.88	449	**227** (85); **127** (100)	30	2.85	240
***6***	**Gentiopicrin ^S,R^**	2.90	415	**179** (60); **119** (100)	20	2.83	250, 280, 370
***4***	**Sweroside ^S,R^**	3.00	417	**195** (100); **179** (85)	20	2.94	250
***3***	**Secologanin ^S,R^**	3.66	447	**155** (100); **123** (30)	30	3.58	240

^S^ Confirmed using reference standards; ^R^ Confirmed according to the literature.

## Supplementary Materials

The following are available online at https://www.mdpi.com/2223-7747/8/12/589/s1, Figure S1: SRM (Single Reaction Monitoring) UHPLC-MS2 chromatograms of targeted compounds (loganic acid, loganin, secologanin, sweroside, swertiamarin and gentiopicrin) and their corresponding MS2 spectra, Figure S2: (**A**) Relative expression of *CeIS1* in *C. erythraea* leaves at determined time points (h) after wounding (HAW). The relative expressions of the *CeIS1* were normalized against *CeEF1* gene as an internal control; unwounded leaves (at 0 h) were set as a calibrator. Since factorial ANOVA revealed that type of leaves does not significantly contribute to the gene expression, only the effect of time was post hoc tested. Bars with different letters are significantly different (*p* < 0.05) according to post hoc Tukey’s test. Abbreviations: IS – iridoid synthase, LL-local leaves, SL-systemic leaves. (**B**) Phylogenetic tree derived from CeIS1, CeIS2 and selected ISs and P5βRs. CLC sequence viewer 8.0 was utilized to align protein sequences and a neighbor joining tree was formed from the alignment. The scale bars represents 0.2 substitutions per site. The GenBank accession numbers are as follows: CrIS (*Catharantus roseus* iridoid synthase, AFW98981.1), CrP5BR4 (*Catharantus roseus* progesterone-5-β-reductase 4, AIW09146.1), GrIS1 (*Gentiana rigescens* iridoid synthase 1, AKI87774.1), GrIS2 (*Gentiana rigescens* iridoid synthase 2, AKI87775.1), DlP5BR1 (*Digitalis lanata* progesterone-5-β-reductase 1, AIF73578.1), DlP5BR2 (*Digitalis lanata* progesterone-5-β-reductase 2, ADL28122.1), DpP5BR2 (*Digitalis purpurea* progesterone-5-β-reductase 2, ACZ66261.1), LjIS (*Lonicera japonica* iridoid synthase, AMB61018.1) and OeIS (*Olea europaea* iridoid synthase, ALV83438.1, Table S1: Primer sequences used for the qPCR analysis (for other SGs metabolic pathway genes primer sets please refer to [4]), Table S2: Results of factorial ANOVA on gene expression data in leaves of *C. erythraea* upon wounding. Factor time included all time points in hours upon wounding (HAW) as in Figure 2 and Figure 3 of the Manuscript. Factor leaf type represents wounded (LL - local leaves) and unwounded leaves (SL - systemic leaves) as indicated in Manuscript. Time x leaf type represents interaction term in factorial ANOVA. Since ANOVA residuals were heteroscedastic in several cases to stabilize the variance Box-Cox transformation was applied prior to ANOVA; this is indicated in the Box-Cox trans column. The asterisks denote the level of statistical significance: * < 0.05, ** < 0.01 and *** < 0.001.
