# Supplementary material for: Secoiridoids Metabolism Response to Wounding in Common Centaury (Centaurium erythraea Rafn) Leaves
Source: Plants (Basel). 2019 Dec 11;8(12):589. doi: 10.3390/plants8120589 (PMC6963686; doi:10.3390/plants8120589)
Supplement: Supplementary file 1 [file plants-08-00589-s001.zip › Supplementary Fig. 1.pdf]

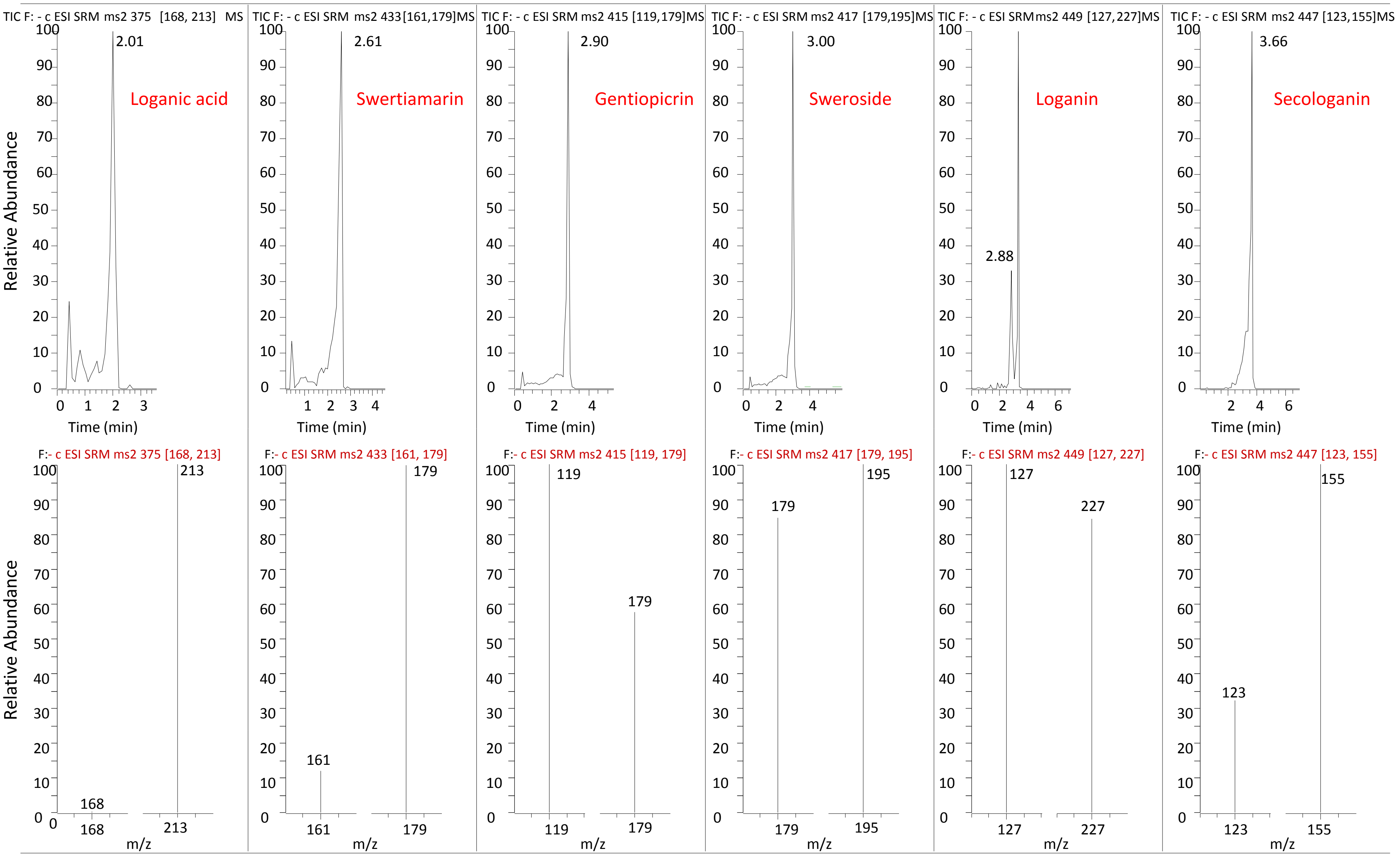

Figure S1. SRM (Single Reaction Monitoring) UHPLC-MS^2 chromatograms of targeted compounds (loganic acid, loganin, secologanin, sweroside, swertiamarin and gentiopicrin) and their corresponding MS^2 spectra.
