# Supplementary material for: Secoiridoids Metabolism Response to Wounding in Common Centaury (Centaurium erythraea Rafn) Leaves
Source: Plants (Basel). 2019 Dec 11;8(12):589. doi: 10.3390/plants8120589 (PMC6963686; doi:10.3390/plants8120589)
Supplement: Supplementary file 1 [file plants-08-00589-s001.zip › Supplementary Fig. 2.pdf]

A

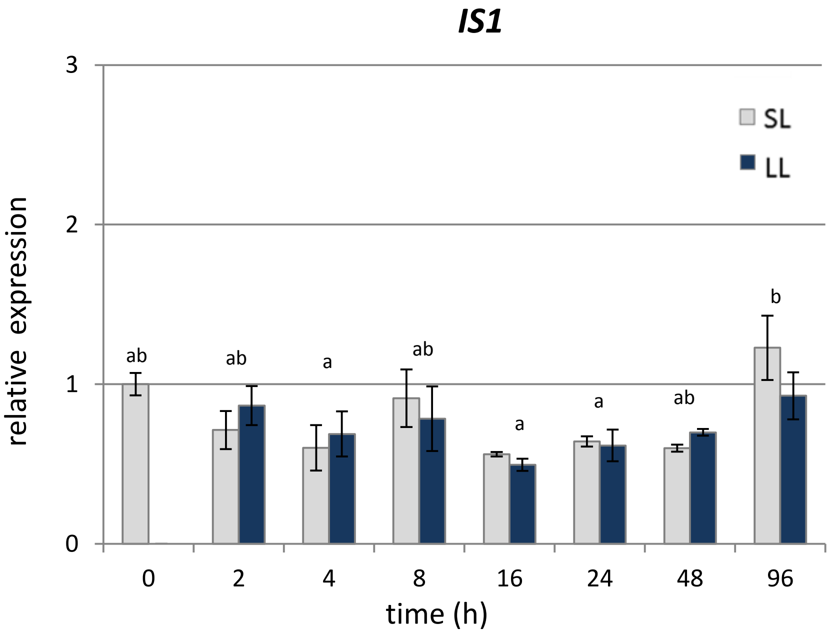

B

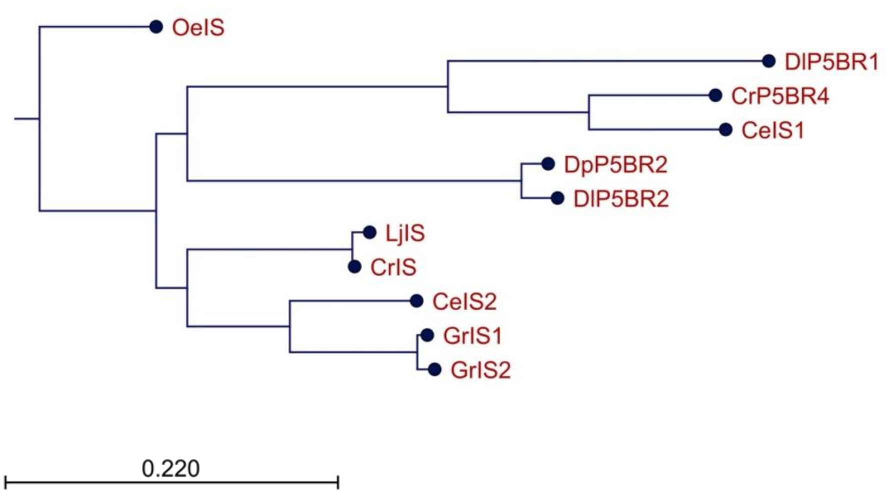

Figure S2. A) Relative expression of *CeIS1* in *C. erythraea* leaves at determined time points (h) after wounding (HAW). The relative expressions of the *CeIS1* were normalized against *CeEF1* gene as an internal control; unwounded leaves (at 0 h) were set as a calibrator. Since factorial ANOVA revealed that condition of leaves does not significantly contribute to the gene expression, only the effect of time was post hoc tested. Bars with different letters are significantly different ($p < 0.05$) according to post hoc Tukey's test. Abbreviations: IS – iridoid synthase, LL-local leaves, SL-systemic leaves. **B)** Phylogenetic tree derived from CeIS1, CeIS2 and selected ISs and P5βRs. CLC sequence viewer 8.0 was utilized to align protein sequences and a neighbor joining tree was formed from the alignment. The scale bars represents 0.2 substitutions per site. The GenBank accession numbers are as follows: CrIS (*Catharantus roseus* iridoid synthase, AFW98981.1), CrP5BR4 (*Catharantus roseus* progesterone-5-β-reductase 4, AIW09146.1), GrIS1 (*Gentiana rigescens* iridoid synthase 1, AKI87774.1), GrIS2 (*Gentiana rigescens* iridoid synthase 2, AKI87775.1), DlP5BR1 (*Digitalis lanata* progesterone-5-β-reductase 1, AIF73578.1), DlP5BR2 (*Digitalis lanata* progesterone-5-β-reductase 2, ADL28122.1), DpP5BR2 (*Digitalis purpurea* progesterone-5-β-reductase 2, ACZ66261.1), LjIS (*Lonicera japonica* iridoid synthase, AMB61018.1) and OeIS (*Olea europaea* iridoid synthase, ALV83438.1).
