# Supplementary material for: Secoiridoids Metabolism Response to Wounding in Common Centaury (Centaurium erythraea Rafn) Leaves
Source: Plants (Basel). 2019 Dec 11;8(12):589. doi: 10.3390/plants8120589 (PMC6963686; doi:10.3390/plants8120589)
Supplement: Supplementary file 1 [file plants-08-00589-s001.zip › Tables S1 S2 S3.pdf]

Table S1. Iridoids identified in methanol extracts of *Centaurium erythraea* by UHPLC/DAD/±HESI-MS^2 analysis. Peak labels, retention times (t_R), parent ions $[M+CH_3COOH+H]^+$ *m/z*, MS^2 fragments used in an SRM (Single Reaction Monitoring) experiment, collision energies (cE), and λ_{max} for each of the compounds are presented.

		**UHPLC-MS data**				**UHPLC-DAD data**	
**Peak No.**	**Assignment**	**t_R (min)**	**$[M+CH_3COOH+H]^-$ [*m/z*]**	**SRM MS^2 fragments [*m/z* (Intensity)]**	**cE (eV)**	**t_R (min)**	**λ_{max} [nm]**
1	**Loganic acid[S,R]**	2.01	**375**	**213** (100), **168** (<5)	20	1.95	240
5	**Swertiamarin[S,R]**	2.61	**433**	**179** (100); **161** (15)	20	2.52	240
2	**Loganin[S,R]**	2.88	**449**	**227** (85); **127** (100)	30	2.85	240
6	**Gentiopicrin[S,R]**	2.90	**415**	**179** (60); **119** (100)	20	2.83	250, 280, 370
4	**Sweroside[S,R]**	3.00	**417**	**195** (100); **179** (85)	20	2.94	250
3	**Secologanin[S,R]**	3.66	**447**	**155** (100); **123** (30)	30	3.58	240

[S]Confirmed using reference standards
[R]Confirmed according to the literature

Table S2. Primer sequences used for the qPCR analysis (for other SGs metabolic pathway genes primer sets please refer to [4])

Genes		Primers (5' to 3')
CeIS2	F	CAAGCCATTGGTGTATCCAG
	R	CACTTGATTCTTCGCCTTGG
CeCOI1	F	CATCAGGAAATGCACACGAT
	R	GTGCCCAGCCAGTGAATAGT
CeJAZ1	F	TTGCTGATTCCGGTAGGTTC
	R	TGAATTTCCTCCACCTGACC
CeBIS1	F	GCTTAATCTTACAGTTACAAGCACAAA
	R	CTACTTGAGTTGGGCGTTGG
CeMYC2	F	CCGAAAAGCAAGACTTGAGG
	R	TGGCTGCTGATGACTTGATG
CeJAM2	F	ATGCAGGGACTTGTCAATGG
	R	AGCCCTCAACATCTGAATGC
CeJAM3	F	TTCTCCGAAGGTGGAAGATG
	R	GCTCCAAGGTAGAAATCATGG
CeEF1	F	AGATGCACCATGAAGCCCTC
	R	GATGACCTGGGAGGTGAAGC

F, forward primer; R, reverse primer

Table S3. Results of factorial ANOVA on gene expression data in leaves of *C. erythraea* upon wounding. Factor **time** included all time points in hours upon wounding (HAW) as in figures 2 and 3 of the Manuscript. Factor **leaf type** represents wounded (LL - local leaves) and unwounded leaves (SL - systemic leaves) as indicated in Manuscript. **Time x leaf type** represents interaction term in factorial ANOVA. Since ANOVA residuals were heteroscedastic in several cases to stabilize the variance Box-Cox transformation was applied prior to ANOVA; this is indicated in the Box-Cox trans column. The asterisks denote the level of statistical significance: * < 0.05, ** < 0.01 and *** < 0.001.

Gene	Box-Cox trans	time	leaf type	time x leaf type
CeGPPS	log	***		
CeGES	log	***		
CeG8O	log	***		
Ce8HGO	sqrt	***		
CeIS2	log	***		
CeIO	none	***		
Ce7DLGT	sqrt	***		
Ce7DLH2	none	***		
CeSLS	sqrt	***		
CeCOI1	sqrt	***		
CeJAZ1	none	***	**	*
CeMYC2	none		*	
CeBIS1	1/sqrt	***		
CeJAM2	none	***		
CeJAM3	none	**		
CeIS1	none	**		
